# Preclinical Characterization of NVR 3-778, a First-in-Class Capsid Assembly Modulator against Hepatitis B Virus

**DOI:** 10.1128/AAC.01734-18

**Published:** 2018-12-21

**Authors:** Angela M. Lam, Christine Espiritu, Robert Vogel, Suping Ren, Vincent Lau, Mollie Kelly, Scott D. Kuduk, George D. Hartman, Osvaldo A. Flores, Klaus Klumpp

**Affiliations:** a Novira Therapeutics, Inc., Spring House, Pennsylvania, USA

**Keywords:** CHB, HBV inhibitors, capsid assembly modulator, chronic hepatitis B

## Abstract

NVR 3-778 is the first capsid assembly modulator (CAM) that has demonstrated antiviral activity in hepatitis B virus (HBV)-infected patients. NVR 3-778 inhibited the generation of infectious HBV DNA-containing virus particles with a mean antiviral 50% effective concentration (EC_50_) of 0.40 µM in HepG2.2.15 cells. The antiviral profile of NVR 3-778 indicates pan-genotypic antiviral activity and a lack of cross-resistance with nucleos(t)ide inhibitors of HBV replication. The combination of NVR 3-778 with nucleos(t)ide analogs *in vitro* resulted in additive or synergistic antiviral activity. Mutations within the hydrophobic pocket at the dimer-dimer interface of the core protein could confer resistance to NVR 3-778, which is consistent with the ability of the compound to bind to core and to induce capsid assembly. By targeting core, NVR 3-778 inhibits pregenomic RNA encapsidation, viral replication, and the production of HBV DNA- and HBV RNA-containing particles. NVR 3-778 also inhibited *de novo* infection and viral replication in primary human hepatocytes with EC_50_ values of 0.81 µM against HBV DNA and between 3.7 and 4.8 µM against the production of HBV antigens and intracellular HBV RNA. NVR 3-778 showed favorable pharmacokinetics and safety in animal species, allowing serum levels in excess of 100 µM to be achieved in mice and, thus, enabling efficacy studies *in vivo*. The overall preclinical profile of NVR 3-778 predicts antiviral activity *in vivo* and supports its further evaluation for safety, pharmacokinetics, and antiviral activity in HBV-infected patients.

## Abstract (precis)

NVR 3-778 is the first capsid assembly modulator (CAM) that has demonstrated antiviral activity in hepatitis B virus (HBV)-infected patients. NVR 3-778 inhibited the generation of infectious HBV DNA-containing virus particles with a mean antiviral 50% effective concentration (EC_50_) of 0.40 µM in HepG2.2.15 cells.

## INTRODUCTION

It is estimated that approximately 250 million patients worldwide are chronically infected with hepatitis B virus (HBV), which is a major cause of chronic liver disease and hepatocellular carcinoma (1, 2). Approved treatments for chronic HBV infection are interferon alpha products or nucleos(t)ide analogs. However, patients rarely achieve functional HBV cure, and most patients who are eligible for treatment are treated with long-term, often life-long administration of nucleos(t)ide analogs (3, 4). Thus, there is a high unmet medical need for new therapies that can deliver improved suppression of virus replication and durable response rates.

HBV is a member of the *Hepadnaviridae* family and carries a genome of relaxed circular DNA (rcDNA) that is converted into covalently closed circular DNA (cccDNA) upon infection of liver cells. Transcription of cccDNA generates pregenomic RNA (pgRNA) and viral messenger RNAs that are translated into viral proteins: precore, core, polymerase, HBV surface antigens (HBsAg), and HBV X protein (HBx). The HBV core protein serves as the building block for forming a capsid structure, which encloses pgRNA and viral polymerase during the capsid assembly process and serves as the site for reverse transcription, leading to the synthesis of viral DNA, and eventually allows the formation of HBV DNA-containing infectious viral particles. Small-molecule compounds that bind to core protein or capsid can interfere with functional capsid assembly or disassembly and often can accelerate capsid assembly in the absence of HBV RNA or polymerase (5–7). Such compounds are also referred to as capsid assembly modulators (CAMs), as they can interfere with pgRNA encapsidation and HBV DNA replication by accelerating or misdirecting the formation of capsid-like structures. In addition to blocking the production of infectious HBV virions, CAMs also block the production of HBV RNA-containing particles in persistently infected cells (8, 9) and can interfere with cccDNA formation when CAMs are present at the time of *de novo* infection (10, 11).

NVR 3-778 is a first-in-class CAM that has shown efficacy in a humanized mouse model and that has completed phase 1 studies in HBV-infected patients (12, 13). Here, we report the preclinical characterization of NVR 3-778, including its antiviral activity against a panel of HBV isolates representing genotypes (GT) A to H and against HBV variants containing clinically relevant reverse transcriptase (rt) mutations that confer resistance to approved anti-HBV nucleos(t)ide analogs. We examined the effect of combining NVR 3-778 with nucleos(t)ide analogs by monitoring the levels of secreted HBV DNA and secreted HBV RNA. The sensitivity of NVR 3-778 against representative core variants with mutations located within the binding pocket was also evaluated. The analysis of the preclinical pharmacokinetics of the compound showed high oral bioavailability and metabolic stability that translated into high serum exposures of NVR 3-778 from oral dosing and guided dose selection for the achievement of *in vivo* efficacy with this compound.

## RESULTS

### NVR 3-778 targets HBV core protein and inhibits viral replication.

**FIG 1:**
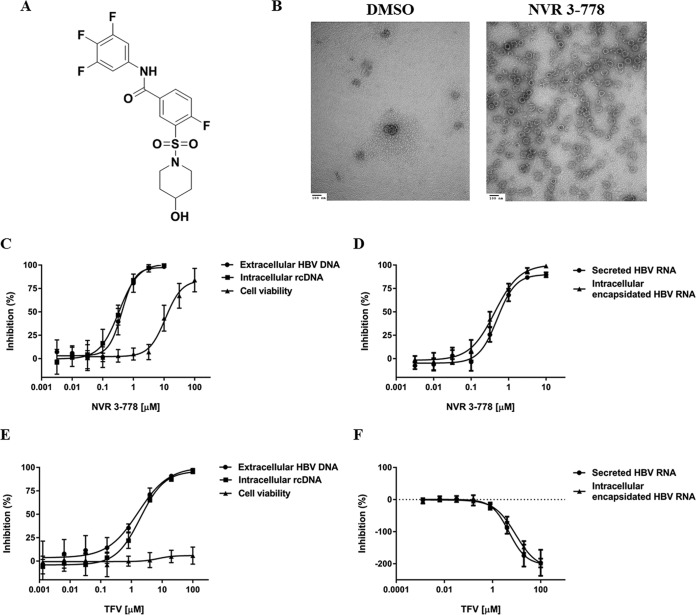
NVR 3-778 targets HBV core protein and inhibits viral replication. (A) Chemical structure of NVR 3-778. (B) Purified recombinant HBV core protein in the absence or presence of NVR 3-778. NVR 3-778 was incubated with recombinant core protein (1.2:1 compound-to-core monomer ratio) in buffer containing 0.15 M NaCl at room temperature overnight prior to staining and imaging by electron microscopy. (C, D) The effect of NVR 3-778 on intracellular rcDNA, extracellular HBV DNA, or cell viability (C) or on intracellular encapsidated pgRNA- or extracellular HBV RNA-containing particles (D) was determined upon treating HepG2.2.15 cells with increasing concentrations of NVR 3-778. (E, F) The effect of TFV on intracellular rcDNA, extracellular HBV DNA, or cell viability (E) or on intracellular encapsidated pgRNA- or extracellular HBV RNA-containing particles (F) was determined upon treating HepG2.2.15 cells with increasing concentrations of TFV. Secreted HBV DNA and secreted HBV RNA levels were determined from the supernatant of HepG2.2.15 cells. Intracellular encapsidated rcDNA and pgRNA levels were determined upon NP-40 lysis of cells and by using S7 nuclease to remove nonencapsidated nucleic acids. Cell viability was determined by measuring ATP levels using the CellTiter-Glo assay. Data points are mean values from at least three independent experiments, with standard deviations shown as error bars.

NVR 3-778 (Fig. 1A) is a small-molecule inhibitor of HBV replication that targets the viral core protein. Under conditions where purified recombinant core protein is mostly in the form of a dimer, the presence of NVR 3-778 effectively induced the formation of capsid-like particles, which can be visualized by electron microscopy (Fig. 1B). Based on this phenotype, the compound is classified as a capsid assembly modulator (CAM). The antiviral activity of NVR 3-778 upon targeting of core was determined using HepG2.2.15 cells (14), which contain stably integrated, replication-competent HBV DNA and generate infectious virions, viral proteins, and HBV RNA-containing particles (8, 15). NVR 3-778 inhibited viral replication (rcDNA formation) and the production of secreted HBV DNA virions in HepG2.2.15 cells with mean 50% effective concentration (EC_50_) values of 0.34 µM and 0.40 µM, respectively (Fig. 1C; Table 1). NVR 3-778 also blocked intracellular HBV RNA encapsidation with a mean EC_50_ value of 0.44 µM and inhibited the production of secreted HBV RNA-containing particles with a similar efficiency (Fig. 1D; Table 1). In contrast, the nucleotide analog tenofovir (TFV) inhibited HBV DNA production (Fig. 1E) and did not inhibit but actually increased the levels of both intracellular encapsidated HBV RNA and secreted HBV RNA in a dose-dependent manner (Fig. 1F).

**TABLE 1:** Effect of NVR 3-778 against HBV and on cell viability using HepG2.2.15 cells^*a*^

Marker	EC_50_ or CC_50_ (µM)		
	Mean	SD	Range
Intracellular encapsidated rcDNA	0.34	0.073	0.26–0.42
Secreted HBV DNA	0.40	0.13	0.10–0.69
Intracellular encapsidated pgRNA	0.44	0.048	0.41–0.49
Secreted HBV RNA	0.60	0.15	0.49–0.77
Cell viability	14.5	5.7	10.0–26.8

a EC_50_ and 50% cytotoxic concentration (CC_50_) values are shown as the mean value and standard deviation from at least three independent studies. Intracellular encapsidated rcDNA and pgRNA were determined by branched DNA and QuantiGene assays, respectively. Cell viability was determined by measuring ATP levels by the CellTiter-Glo assay.

**FIG 2:**
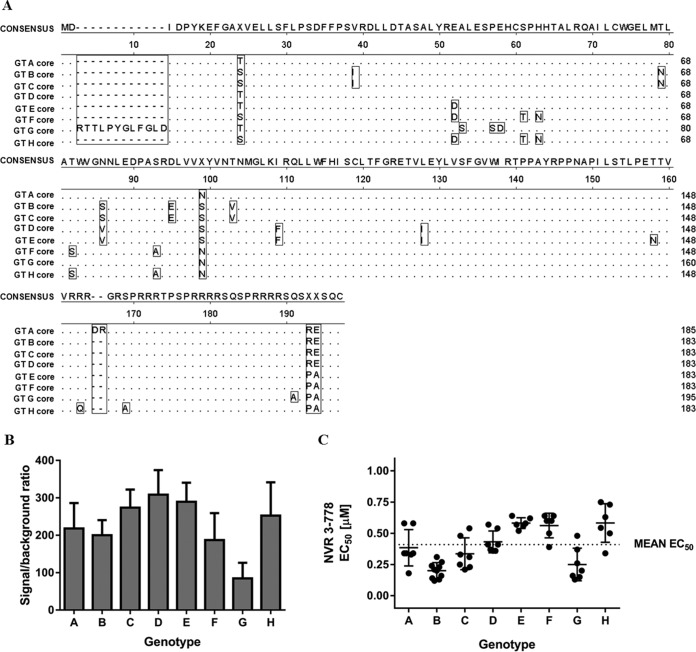
Transient-transfection studies using representative HBV strains from GT A to H. (A) Amino acid sequence alignment of HBV core encoded in the viral genomes from GT A to H isolates used in the transient-transfection studies. (B) Relative replication capacities of HBV of GT A to H. HepG2 cells were transfected with HBV-containing plasmids. HBV DNA replication was determined at 3 days posttransfection by measuring intracellular encapsidated HBV DNA signals. Data represent the mean values from at least three independent transfection experiments, and standard deviations are shown as error bars. (C) HepG2 cells transfected with HBV-containing plasmids were treated with increasing concentrations of NVR 3-778 for 3 days. Intracellular encapsidated HBV DNA levels were monitored and compared with those for untreated cells. Data points are EC_50_ values from independent experiments. Means and standard deviations are shown.

HBV can be classified into genotypes (GT) A to J, which can be associated with the geographical distribution and differentiated clinical implications, including liver cancer development and treatment responses (16). Our previous *in vivo* efficacy study using humanized mice infected with GT C HBV showed that NVR 3-778 was effective at reducing both serum HBV DNA and HBV RNA levels (13). Here, using plasmids that contained 1.1 times the genome length of the HBV genomes under the control of a cytomegalovirus (CMV) promoter, we evaluated the antiviral activity of NVR 3-778 against a panel of representative HBV strains from the eight major genotypes (GT A to H) (the GenBank sequences are summarized in Table S1 in the supplemental material). Pairwise nucleotide sequence alignment of the full-length viral genomes indicated that the identities of the sequences in this panel ranged from 86% for the most distantly related HBV isolates to 92% for the most closely related HBV isolates. Alignment of the HBV core protein showed a high degree of conservation, but of note was that core from GT B, C, D, E, F, and H isolates contained 183 amino acids, while core from GT A isolates contained 2 additional amino acids within the C-terminal domain and core from GT G isolates contained 12 additional amino acids at the N terminus (Fig. 2A).

HepG2 cells were transfected with each of the plasmids containing HBV GT A to H, and intracellular encapsidated HBV DNA levels were monitored to determine the replication capacity among the various genotypes. Compared to untransfected HepG2 cells in which the HBV DNA signal-to-background ratio was set to 1, cells transfected with GT G HBV produced intracellular encapsidated HBV DNA with a mean signal-to-background ratio of 85, while the other genotypes achieved mean signal-to-background ratios of above 100 (Fig. 2B), indicating that these HBV isolates were replicative and suitable for antiviral studies. Treatment of transfected HepG2 cells with NVR 3-778 showed that the compound was active across GT A to H HBV isolates, inhibiting viral replication with mean EC_50_ values that ranged from 0.20 µM to 0.58 µM, with the lowest EC_50_ being observed for the GT B isolate and the highest EC_50_ being observed for the GT E and GT H isolates (Fig. 2C; Table 2). Overall, the antiviral activity of NVR 3-778 against these representative HBV isolates was similar to the antiviral activity observed in HepG2.2.15 cells with a stably integrated HBV genome of GT D from a different isolate.

**TABLE 2:** Antiviral activities of NVR 3-778 in HepG2 cells transiently transfected with plasmids containing representative HBV isolates from GT A to H^*a*^

HBV GT	EC_50_ (µM)			No. of expt
	Mean	SD	Range	
A	0.38	0.15	0.18–0.58	7
B	0.20	0.064	0.12–0.31	10
C	0.34	0.13	0.21–0.54	7
D	0.43	0.086	0.36–0.57	7
E	0.58	0.044	0.52–0.64	6
F	0.56	0.10	0.39–0.64	6
G	0.25	0.13	0.13–0.48	7
H	0.58	0.15	0.34–0.75	6

a Intracellular encapsidated rcDNA levels were monitored and compared with those in untreated cells. Mean EC_50_ values and standard deviations were determined from multiple independent experiments, as indicated.

### Nucleos(t)ide-resistant HBV variants remain sensitive to inhibition by NVR 3-778.

The most common therapy for chronic hepatitis B is long-term treatment with nucleos(t)ide analogs as antiviral agents to reduce the production of infectious HBV DNA-containing particles. Such long-term, typically multiyear treatment with a single nucleos(t)ide analog can result in the selection of drug-resistant HBV variants that carry mutations within the viral reverse transcriptase (rt) protein. New drugs or drug combinations that are active against nucleos(t)ide-resistant virus variants could, in the future, prevent the incidence of resistance selection or provide a treatment option for patients with drug-resistant HBV. We tested the antiviral activity of NVR 3-778 against a panel of reverse transcriptase variants with amino acid changes identified from patients who developed resistance to nucleos(t)ide analogs (17, 18). The panel consisted of variants with either single, double, or triple mutations: (i) rtL180M/M204V, (ii) rtN236T, (iii) rtA181V, (iv) rtA181V/N236T, and (v) rtL180M/M204V/N236T. These variants were evaluated by the transient transfection of plasmids carrying the replication-competent virus variants described above. Variants with single mutations of rt, the rtN236T and rtA181V variants, replicated at approximately 77% and 143% of the capacity of wild-type (WT) HBV, respectively; the L180M/M204V variant replicated about 2-fold better than the WT, and introducing the A181V or L180M/M204V mutations together with N236T compensated for the reduced replication capacity of the N236T variant (Fig. S1A).

Both the rtL180M/M204V and rtL180M/M204V/N236T variants were resistant to inhibition by lamivudine (LMV) and entecavir (ETV): LMV inhibited WT HBV with a mean EC_50_ of 0.53 µM but did not inhibit the two variants up to the highest concentration tested (100 µM), while the antiviral activity of ETV (WT HBV EC_50_, 0.0014 µM) against the rtL180M/M204V and rtL180M/M204V/N236T variants was reduced by 31- and 14-fold, respectively (Table 3; Fig. S1D and E). Tenofovir disoproxil fumarate (TDF) showed similar antiviral activity against the WT and the variants with the rtL180M/M204V and rtA181V mutations (mean EC_50_ values, 0.032, 0.034, and 0.043 µM, respectively) but showed a moderate reduction of antiviral activity (about 3-fold) against the variants that contained rtN236T (Table 3; Fig. S1F), similar to previously reported data (19). HBV containing the single rtN236T mutation remained sensitive to inhibition by LMV and ETV, but combining the rtN236T mutation with the rtA181V mutation resulted in a mean 4.8-fold increase in the EC_50_ value for LMV (Table 3). All five nucleoside-resistant HBV variants remained sensitive to inhibition by NVR 3-778 (Fig. S1B and C), with the mean fold changes in the EC_50_ from that obtained for the WT ranging from 0.82 to 1.4 (Table 3).

**TABLE 3:** Antiviral activity of NVR 3-778, LMV, ETV, and TDF in HepG2 cells transiently transfected with nucleoside-resistant HBV variants

Compound	WT EC_50_ (µM)	EC_50_ fold change^*c*^				
		rtL180M/M204V	rtL180M/M204V/N236T	rtA181V	rtN236T	rtA181V/N236T
LMV	0.53 ± 0.12	>190	>190	1.7 ± 0.9^*d*^	1.0 ± 0.5^*d*^	4.8 ± 2.3^*a*^
ETV	0.0014 ± 0.0004	31 ± 16^*a*^	14 ± 4^*a*^	2.2 ± 0.5^*a*^	0.67 ± 0.22^*d*^	1.8 ± 0.6^*d*^
TDF	0.032 ± 0.015	1.1 ± 0.3^*d*^	2.9 ± 1.5^*b*^	1.4 ± 0.05^*d*^	2.2 ± 1.0^*b*^	2.8 ± 1.4^*b*^
NVR 3-778	0.31 ± 0.10	1.3 ± 0.6^*d*^	1.4 ± 0.5^*d*^	0.82 ± 0.19^*d*^	0.85 ± 0.40^*d*^	0.85 ± 0.26^*d*^

a The *t*-test *P* value was <0.01.

b The *t*-test *P* value was <0.05.

c Ratio of the mean EC_50_ value for the HBV inhibitors determined against reverse transcriptase variants over those against wild-type (WT) HBV. EC_50_ fold change values compared to the value for the WT are shown as the mean value ± standard deviation from at least three independent studies.

d The *t*-test *P* value was not significant (*P* > 0.05).

### Combination effect of NVR 3-778 and nucleos(t)ide analogs.

**FIG 3:**
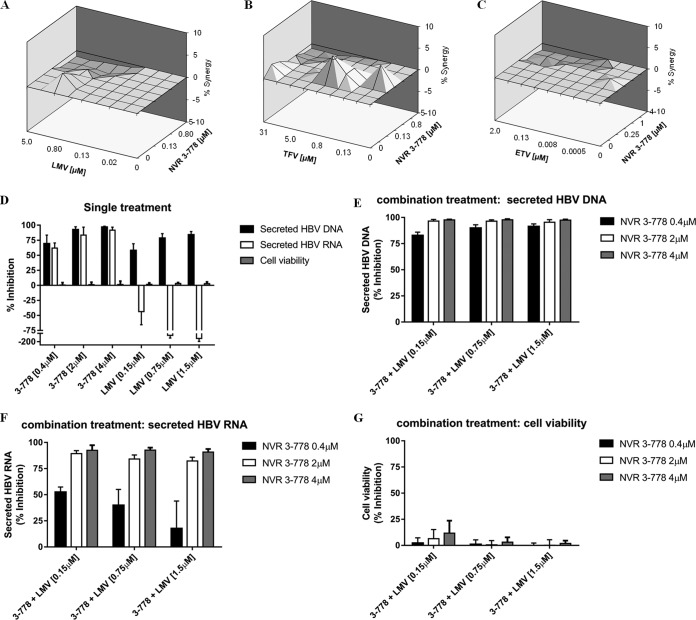
Effect of NVR 3-778 in combination with nucleos(t)ide analogs. HepG2.2.15 cells were treated with increasing concentrations of NVR 3-778, LMV, TFV, or ETV for 6 days. (A to C) Synergy plots at 95% confidence calculated from MacSynergy II software. Three different assay plates of HepG2.2.15 cells treated with increasing concentrations of NVR 3-778 in combination with LMV (A), TFV (B), or ETV (C) in a checkerboard format were used. (D to G) The effect of combining NVR 3-778 and a nucleoside analog on secreted HBV RNA was further evaluated. HepG2.2.15 cells were treated for 6 days with NVR 3-778 (0.4 μM, 2 μM, and 4 μM) or LMV (0.15 μM, 0.75 μM and 1.5 μM) either alone or in combination. (D) The effect of NVR 3-778 or LMV on secreted HBV DNA, secreted HBV RNA, or cell viability in cells receiving treatment with a single drug. (E to G) Effect of combining NVR 3-778 and LMV on secreted HBV DNA (E), secreted HBV RNA (F), and cell viability (G). Each concentration of NVR 3-778 (0.4 μM, 2 μM, and 4 μM) was combined with LMV dosed at 0.15 μM, 0.75 μM, or 1.5 μM. Extracellular HBV DNA and HBV RNA levels were determined from supernatants and compared to those for untreated cells. Cell viability was determined by measuring ATP levels using the CellTiter-Glo assay. Data points represent mean values, and standard deviations are shown as error bars.

The antiviral effect of combining NVR 3-778 and nucleos(t)ide analogs (LMV, TFV, or ETV) was also examined, and the results were analyzed using MacSynergy II and CalcuSyn software. HepG2.2.15 cells were treated with increasing concentrations of NVR 3-778 (up to 4 μM) either alone or in combination with increasing concentrations of ETV (up to 2 μM), LMV (up to 5 μM), or TFV (up to 31.25 μM). According to MacSynergy analysis at 95% confidence, combining NVR 3-778 with each of the three nucleoside analogs showed an additive antiviral effect (Fig. 3A to C; Table 4). Similar results were obtained from the CalcuSyn analysis (Table 4). The effect of the compound combinations on cell viability was evaluated by measuring the intracellular ATP levels of HepG2.2.15 cells. The results showed that cell viability remained above 90% in samples treated with the highest compound concentrations either alone or in combination.

**TABLE 4:** Synergy/antagonism analysis for NVR 3-778 in combination with nucleoside analogs using MacSynergy and CalcuSyn

Analog used in combination with NVR 3-778	Synergy (μM^2^%)	Antagonism (μM^2^%)	MacSynergy-predicted effect^*a*^	CI value^*b*^			Overall CI^*c*^	CalcuSynb-predicted effect^*d*^
				ED_50_	ED_75_	ED_90_		
LMV	5.1	−14.3	Additive	1.0	0.8	0.8	0.9 ± 0.1	Additive
TFV	18.5	−7.3	Additive	0.8	0.8	0.8	0.8 ± 0.06	Slight to moderate synergy
ETV	1.0	−16.5	Additive	1.0	0.5	0.5	0.7 ± 0.4	Slight to moderate synergy

a Synergy/antagonism volumes at 95% confidence of <25 µM^2^% were defined as insignificant, those between 25 and 50 µM^2^% were defined as minor, those between 50 and 100 µM^2^% were defined as moderate, and those of >100 µM^2^% were defined as strong.

b CI, confidence interval. The confidence intervals at ED_50_, ED_75_, and ED_90_ represents mean values determined from at least 5 different combination ratios.

c Mean and standard deviation of all confidence interval (CI) values.

d The combination effect prediction based on overall confidence interval values is as follows: <0.7 is synergy, 0.7 to 0.9 is slight to moderate synergy, 0.9 to 1.1 is additive, 1.1 to 1.5 is slight to moderate antagonism, and >1.5 is antagonism.

The nucleos(t)ide analogs and NVR 3-778 inhibit viral replication by different mechanisms and by targeting different viral proteins. As shown above and as previously published, while the HBV CAMs inhibited the formation of both HBV DNA and HBV RNA-containing particles, the nucleos(t)ide analogs only suppressed viral replication but increased the production of HBV RNA-containing particles in HepG2.2.15 cells and infected hepatocytes (8, 9). We therefore investigated the outcome of combining these inhibitor classes on the secreted HBV RNA endpoint. We treated HepG2.2.15 cells with three different concentrations of NVR 3-778 and LMV starting at 0.4 μM and 0.15 μM, respectively, which corresponded to their respective EC_50_ values against HBV DNA replication in HepG2.2.15 cells. Consistent with the MacSynergy and CalcuSyn analyses, both NVR 3-778 and LMV inhibited HBV DNA with increasing concentrations, and their combination further increased the inhibitory effect of HBV DNA (Fig. 3D and E). In contrast, treatment with increasing concentrations of LMV alone increased the production of secreted HBV RNA in a dose-dependent manner, while treatment with NVR 3-778 alone inhibited the production of secreted HBV RNA (Fig. 3D). Notably, the addition of 0.4 μM NVR 3-778 to LMV partially reversed the increase in the level of secreted HBV RNA induced by LMV (Fig. 3F), while 2 μM and 4 μM NVR 3-778 completely reverted the LMV effect and resulted in maximal inhibition of secreted HBV RNA to levels similar to those achieved by NVR 3-778 alone (Fig. 3F). Treating cells with NVR 3-778 and LMV either alone or in combination did not affect cell viability (Fig. 3D and G).

### Susceptibility of naturally existing core variants to NVR 3-778.

The binding site of CAMs on the HBV core protein has been determined at a high resolution by crystallography (5, 20). Based on the analysis of the HBV sequences in GenBank, residues 102, 105, 109, and 118 of the core protein were identified to be polymorphic, defined as residues with variants occurring at frequencies above 1% either across genotypes or within a particular genotype (5). The replication competence of core variants at these positions was previously determined: the W102G and W102R variants were incompetent for HBV replication, the Y118F variant replicated at a level of about 45% of that of the WT, while the I105 and T109 variants replicated either at levels similar to or at a level about 70% of that of the WT HBV (5). HBV CAMs from the heteroarylpyrimidine (HAP) chemical class target core through binding within the dimer-dimer interface and in the process misdirect capsid assembly and induce the formation of aggregated protein structures (5, 7). Site-directed mutagenesis studies showed that core variants with the T109I, T109M, or Y118F amino acid change were resistant to Bay 41-4109 and NVR_010_001_E2 (both HAPs), but variants with mutations at the I105 position remained susceptible to these two compounds (Table 5). The EC_50_ values of NVR_010_001_E2 and Bay 41-4109 against the Y118F mutant were increased by 3.7- and 8.2-fold, respectively. The EC_50_ values of NVR_010_001_E2 for the T109M and T109I mutants were increased by 2.8- and 9.2-fold, respectively. Bay 41-4109 also showed reduced antiviral activities against the T109M and T109I mutants, with mean 3.7- and 21-fold increases in EC_50_ values, respectively.

**TABLE 5:** Antiviral activities of NVR 3-778 and LMV against wild-type HBV and HBV core protein variants

HBV variant	NVR 3-778 EC_50_ (µM)^*a*^	EC_50_ FC^*b*^			LMV EC_50_ (µM)^*a*^	LMV EC_50_ FC^*b*^
		NVR 3-778	NVR-010-001-E2^*c*^	Bay 41-4109^*c*^		
Wild type	0.23 ± 0.11	1	1	1	0.26 ± 0.18	1
Y118F	1.7 ± 0.7	7.4	3.7	8.2	0.33 ± 0.22	1.3
T109S	0.77 ± 0.27	3.3	0.43	0.40	0.22 ± 0.080	0.96
T109M	0.28 ± 0.13	1.2	2.8	3.7	0.22 ± 0.10	0.96
T109I	0.15 ± 0.061	0.65	9.2	21	0.31 ± 0.17	1.2
I105L	0.34 ± 0.13	1.5	0.49	0.44	0.35 ± 0.19	1.3
I105T	1.5 ± 0.3	6.5	1.2	1.5	0.26 ± 0.056	1.0
I105V	0.41 ± 0.11	1.8	1.3	1.3	0.22 ± 0.063	0.84

a Mean EC_50_ values and standard deviations from at least three independent studies determined in HepG2 cells.

b FC, fold change. The EC_50_ fold change refers to the ratio of the mean EC_50_ value for the core variants over that for wild-type HBV.

c Previously published results (5) for comparison with NVR 3-778.

**FIG 4:**
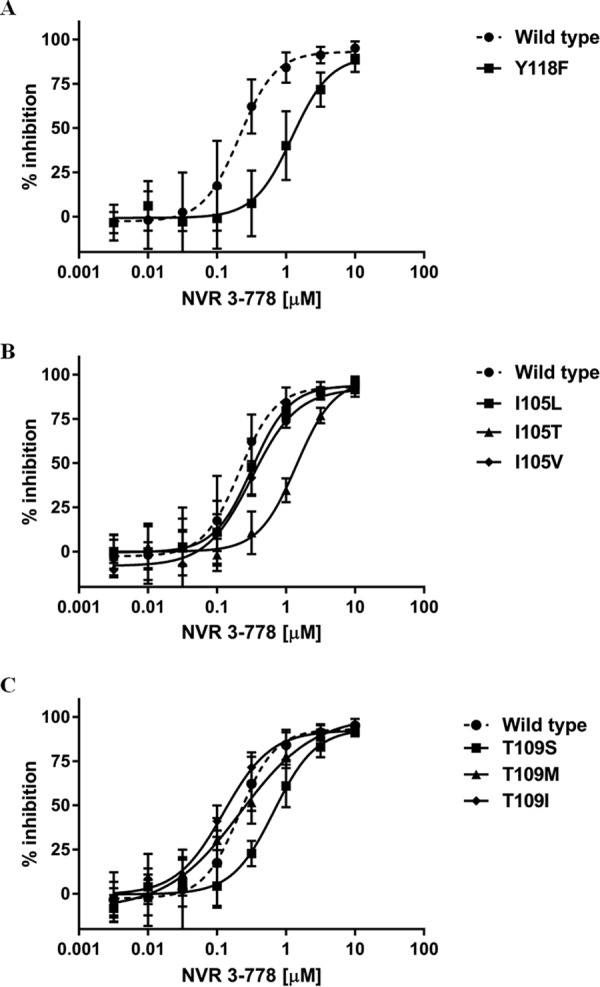
Susceptibility of HBV core variants to inhibition by NVR 3-778. HepG2 cells transiently transfected with wild-type HBV or the Y118F core variant (A), the I105L, I105T, or I105V core variant (B), or the T109S, T109M, or T109I core variant (C) were incubated with increasing concentrations of NVR 3-778 for 3 days. Intracellular encapsidated HBV DNA levels were monitored and compared with those for untreated cells. The dose-response curves for NVR 3-778 against HBV containing the wild-type genome are shown as dashed lines. Data points represent mean values from at least three independent antiviral studies, and standard deviations are shown as error bars.

Here we examined the antiviral activity of NVR 3-778 against HBV variants with amino acid changes at positions 105, 109, and 118 within the core protein (Table 5). NVR 3-778 showed lower antiviral activity against the Y118F variant, with a mean 7.4-fold increase in the EC_50_ compared to that for WT HBV (Fig. 4A). NVR 3-778 maintained its antiviral activity against the I105L and I105V variants, but the compound was less active against the I105T variant, with a mean increase in the EC_50_ value of 6.5-fold compared to that for the WT (Fig. 4B). Contrary to the profile of the HAPs, NVR 3-778 maintained its antiviral activity against the T109M and T109I variants, but the compound was moderately less active against the T109S variant, with a mean increase in the EC_50_ value of 3.3-fold (Fig. 4C).

Importantly, each of these core variants remained fully susceptible to the inhibitory effect of the nucleoside analog LMV, with mean EC_50_ fold changes of 0.84 to 1.3 compared to the EC_50_ for WT HBV (Table 5).

### NVR 3-778 suppresses HBV antigens and HBV RNA production during *de novo* infection.

The establishment of *in vitro* cell-based natural infection systems using primary human hepatocytes (PHH) enabled the study of antiviral compounds during the early steps of the HBV life cycle. In particular, HBV CAMs, but not nucles(t)ide analogs, prevented the formation of cccDNA and the subsequent production of HBV RNA and viral antigens when the compound was added together with the viral inoculum when infecting PHH (10, 21). The effect of NVR 3-778 during *de novo* infection was examined by adding increasing concentrations of the compound together with the HBV inoculum prior to infecting PHH (Fig. S2A). LMV was used as a nucleoside analog control in this assay. PHH from three different donors were used, and the results showed that, in addition to inhibiting HBV DNA particle production (EC_50_, 0.81 μM), NVR 3-778 also inhibited the production of HBsAg, HBeAg, and intracellular total HBV RNA with mean EC_50_ values of 4.8, 4.5, and 3.7 μM, respectively (Table 6; Fig. S2B). The effect of NVR 3-778 on host gene transcription was monitored by measuring the level of intracellular β-actin mRNA as a constitutively expressed housekeeping gene. Inhibition of β-actin mRNA by NVR 3-778 was observed only at the highest concentration tested (30 μM). In contrast, LMV effectively inhibited HBV DNA particle production, but the nucleoside analog was unable to inhibit the production of HBsAg, HBeAg, or HBV RNA when added at the time of HBV infection (Table 6; Fig. S2C).

**TABLE 6:** Antiviral activities of NVR 3-778 and LMV in PHH during *de novo* infection^*a*^

Molecule	NVR 3-778		LMV	
	EC_50_ (µM)	EC_90_ (µM)	EC_50_ (µM)	EC_90_ (µM)
HBV DNA	0.81 ± 0.18	5.4 ± 2.0	0.016 ± 0.010	0.066 ± 0.010
HBsAg	4.8 ± 0.98	10.2 ± 3.0	>30	>30
HBeAg	4.5 ± 0.82	8.9 ± 1.2	>30	>30
Intracellular HBV RNA	3.7 ± 0.22	11.6 ± 2.7	>30	>30
β-Actin mRNA	10 μM < CC_50_ <30 μM^*b*^	10 μM < CC_90_ <30 μM^*b*^	>30	>30

a Mean EC_50_ values and standard deviations were determined using PHH from three different donors. CC_50_, 50% cytotoxic concentration.

b Inhibition of β-actin mRNA by NVR 3-778 was observed only at the highest concentration tested (30 μM).

### Pharmacokinetics of NVR 3-778 in mice.

Fed male and female CD1 mice (36/sex/group) were dosed twice a day (BID) by oral gavage with 50, 150, and 500 mg/kg of body weight/dose of NVR 3-778 for 28 days. At different time points after dosing, plasma was analyzed for NVR 3-778 concentration by liquid chromatography-tandem mass spectrometry (LC-MS/MS). All plasma concentrations in samples from the control group animals were below the limit of detection. The pharmacokinetic parameters are listed in Table 7. Exposure (maximum plasma concentration [*C*_max_] and the area under the concentration-time curve [AUC]) to NVR 3-778 increased with the dose level but did so less than dose proportionally. Gender differences in NVR 3-778 exposure were less than 2-fold, and exposures were generally similar on day 1 and day 28. Mice reached mean *C*_max_ values of NVR 3-778 above 100 µM (43.2 µg/ml) in this 28-day study when dosed orally at 500 mg BID. When dosed at 405 mg/kg BID to uPA/SCID mice with humanized chimeric livers, the mean *C*_max_ levels of NVR 3-778 were 73 to 87 µM, similar to the prediction from the CD1 mice. The mean predose trough concentrations of NVR 3-778 in the humanized mice were 28 to 31 µM, and under these conditions, NVR 3-778 showed antiviral activity similar to that of the nucleoside analog ETV in HBV-infected humanized mice (13).

**TABLE 7:** Mean pharmacokinetic parameters for NVR 3-778 in mouse plasma following twice-daily oral gavage for up to 28 days^*b*^

Day	Dose level (mg/kg/dose)	Sex	*C*_max_ (ng/ml)	*T*_max_^*a*^ (h)	AUC_0–12_ (ng⋅h/ml)	*t*_1/2_ (h)
1	50	M	12,700	1.00	70,400	NC
		F	11,700	0.500	60,100	2.20
	150	M	25,300	1.00	166,000	5.00
		F	22,000	0.500	131,000	4.35
	500	M	43,000	1.00	406,000	NC
		F	41,400	0.500	315,000	NC
28	50	M	11,900	3.00	87,300	NC
		F	11,500	3.00	78,800	NC
	150	M	24,200	1.00	157,000	NC
		F	26,600	1.00	171,000	2.48
	500	M	43,800	3.00	375,000	3.15
		F	40,800	3.00	354,000	NC

a Time is relative to the second daily dose.

b F, female; M, male; AUC_0–12_, area under the concentration-time curve from 0 to 12 h after the second daily dose; *C*_max_, maximum plasma concentration (the lower limit of quantification was 65.0 ng/ml); NC, not calculable; *t*_1/2_, half-life; *T*_max_, time to maximum plasma concentration.

### Oral bioavailability of NVR 3-778 in dogs.

The oral bioavailability of NVR 3-778 was studied following a single intravenous (i.v.) (0.5 mg/kg) and a single oral gavage (1.5 mg/kg) administration to two male beagle dogs with at least a 7-day washout between doses. Following i.v. administration, NVR 3-778 showed a low plasma clearance with a mean value of 6.1 ml/min/kg and a moderate volume of distribution at steady state (*V*_ss_) with a mean value of 1.7 liters/kg. The mean terminal half-life and AUC from 0 h to infinity (AUC_0–inf_) were 4.1 h and 1.38 µg·h/ml, respectively. Following oral administration, the mean *C*_max_ and AUC_0–inf_ values were 0.56 µg/ml and 3.50 µg·h/ml, respectively. The mean oral bioavailability was determined to be 84.6%.

### Hepatocyte stability and metabolism of NVR 3-778 in hepatocytes.

Following *in vitro* incubation for 120 min with human, mouse, or dog hepatocytes, >85% of the NVR 3-778 remained intact. A similar result was demonstrated in the plasma of dogs and mice administered NVR 3-778 by oral gavage, where the NVR 3-778 parent compound was the predominant circulating molecule. The fewest number of metabolites was found following incubation with human hepatocytes, where 92% of the NVR 3-778 remained after a 120-min incubation.

The binding of NVR 3-778 to the plasma proteins of mouse, rat, dog, monkey, and human was investigated by equilibrium dialysis. At the tested concentration of 2 µM, the mean percent binding ranged from approximately 91% for rat to approximately 98% for human, demonstrating that NVR 3-778 is highly protein bound.

To determine the effect of human protein binding on NVR 3-778 antiviral activity, HepG2.2.15 cells were incubated with increasing concentrations of NVR 3-778 in the presence of 0% to 40% human serum. The EC_50_ values of NVR 3-778 were increased by 4.5-, 9.3-, and 15.8-fold in the presence of 10%, 20%, and 40% human serum, respectively (Table 8; Fig. S3).

**TABLE 8:** Effect of human serum on NVR 3-778 antiviral activities

Human serum (%)	EC_50_ (µM)	EC_50_ fold shift^*a*^	EC_90_ (µM)	EC_90_ fold shift^*a*^
0	0.40 ± 0.10	1	1.5 ± 0.41	1.0
10	1.8 ± 0.51	4.5	6.9 ± 0.9	4.5
2	3.7 ± 1.0	9.3	15.9 ± 6.3	10.3
40	6.3 ± 2.0	15.8	23.5 ± 11.6	15.2

a Ratio of the mean EC_50_ and EC_90_ values in the presence of human serum compared to those without human serum. The EC_50_ and EC_90_ values shown are the mean value ± standard deviation from at least three independent studies.

## DISCUSSION

Current antiviral therapies available to chronic hepatitis B patients rarely achieve functional cure, and patients are still at risk of developing severe liver diseases, including hepatocellular carcinoma. One strategy to improve the cure rate is to intensify the suppression of viral replication, and recent research in developing small-molecule compounds indicates that interfering with core protein function can trigger multiple effects within the viral life cycle (8, 10, 11, 21). HBV capsid assembly modulators (CAMs) bind to HBV core and capsid, and compounds from different chemical series are currently being evaluated in preclinical and clinical studies. NVR 3-778 was the first representative of the CAM class of compounds that entered clinical trials in HBV-infected patients and has shown significant antiviral activity alone and in combination with pegylated interferon alpha (pegIFN) (22). Here, our preclinical characterization shows that by targeting core, NVR 3-778 induces capsid assembly and, in the process, suppresses pgRNA encapsidation, viral replication, and the production of both HBV DNA- and HBV RNA-containing particles. The blockage of HBV RNA-containing particle production by HBV CAMs, but not nucleos(t)ide analogs, is a direct result of interfering with pgRNA encapsidation and has been reported for different chemical classes of CAMs (8, 9). Another important differentiation factor is that CAMs, but not nucleos(t)ide analogs, can block cccDNA formation during *de novo* infection and the subsequent steps of transcription and viral protein translation (10, 11, 21). Our data showed that NVR 3-778 suppressed HBsAg, HBeAg, and intracellular HBV RNA production when added to primary human hepatocytes at the time of infection. The EC_50_ values for the inhibition of HBV antigen and HBV RNA formation were 4.6- to 5.9-fold higher than the EC_50_ value for the inhibition of HBV DNA replication in the *de novo* infection experiments. Other studies evaluating CAMs during the early steps of infection have also noted shifts in effective concentrations between HBV DNA and viral antigens and total HBV RNA (10, 21). While the exact mechanism of how CAMs block cccDNA formation is not yet clear, studies using purified nucleocapsids suggest that CAMs could affect the proper disassembly of preformed nucleocapsids and the stability of the encapsidated rcDNA (11). Higher compound concentrations may be needed to interfere with the functional disruption of preformed nucleocapsids, which consist of oligomers of core dimers, while lower concentrations of CAMs may be sufficient to interfere with the formation of capsids from individual dimers. It may also be of interest to investigate, in future studies, if the binding of CAMs, such as NVR 3-778 (which induce the formation of capsid-like particles or empty capsids), and compounds from the HAP chemical family (which induce the formation of enlarged and aggregated misassembled capsids) could affect the proteolytic processing of core and capsid protein and the subsequent presentation of specific HBcAg epitopes on major histocompatibility complex class I and class II molecules.

Biochemical studies using purified recombinant core protein showed that the interaction of NVR 3-778 with core accelerated the formation of capsid-like particles. An independently performed study used compound SBA_R01, which has the same chemical structure as NVR 3-778, to generate a high-resolution cocrystal structure with HBV core protein (23). This study confirmed that the binding site of NVR 3-778 is the HBV core dimer-dimer interface, consistent with the ability of the compound to induce core protein assembly *in vitro*. The binding site was located within a defined hydrophobic pocket at the dimer-dimer interface, which was also targeted by NVR_010_001_E2 and other CAMs that belong to the HAP chemical series (5, 20, 23). Examination of amino acids close to the ligand binding site showed that while NVR 3-778 and compounds from the HAP series target the same pocket, the interaction surfaces overlap but are not identical. We used a panel of HBV variants with mutations in the core protein-coding sequence that represent naturally existing polymorphisms in the binding site to compare the impact of the molecular interaction differences on the phenotype of HBV replication inhibition. While NVR 3-778 remained similarly active against the T109M and T109I variants as against wild-type virus, it was less active against HBV with I105T and Y118F changes in the core protein. Previously, we reported that CAMs from the HAP family remained active against the I105T variant but showed reduced activities against the Y118F, T109M, and T109I variants (5). These results indicate that CAMs from different chemical series could have different resistance profiles *in vitro*, consistent with their differences in binding site interactions. Others have also reported that certain amino acids within the core binding site conferred resistance (e.g., T33N and P25G), while other amino acid changes showed a lack of cross-resistance (e.g., V124F) against CAMs from different chemical classes (23). It will therefore be important to determine the individual resistance profiles for different CAM compounds to better understand and predict the cross-resistance potential between CAM compounds and compound classes. Interestingly, it was recently reported that a patient with a preexisting T109M core variant showed a reduced antiviral response to ABI-H0731, an HBV CAM currently in phase 1b clinical development (24). When both the *in vitro* and *in vivo* observations are taken together, these data suggest that core variants with changes within the ligand binding site can impact the antiviral response of HBV CAMs.

Notably, HBV variants with mutations in the core protein that were less sensitive to inhibition by CAMs remained fully susceptible to the antiviral activity of the nucleos(t)ide analogs. Also, HBV variants with mutations in the viral polymerase that conferred resistance to the nucleos(t)ide analogs remained sensitive to inhibition by core protein-targeting CAMs. It may therefore be beneficial to combine CAMs and nucleos(t)ide analogs to increase antiviral replication suppression and reduce the rate of resistance selection. *In vitro* combination studies with NVR 3-778 and nucleos(t)ide analogs showed additive or synergistic effects in reducing both HBV DNA replication and the production of HBV RNA particles, supporting the premise that the combination treatment with antiviral regimens with different modes of action could further intensify the suppression of HBV replication. Indeed, using HBV-infected humanized mice as an *in vivo* efficacy model, we observed a greater reduction in both serum HBV DNA and HBV RNA levels in mice that were treated with both NVR 3-778 and pegIFN as combination treatment than in mice that were treated with NVR 3-778 alone, pegIFN alone, or entecavir alone (13). This result translated to clinical observations in HBV-infected patients in which the greatest antiviral effect was observed when NVR 3-778 was combined with pegIFN than when NVR 3-778 monotherapy was used (22).

Using a panel of HBV isolates representing GT A to H, we determined that the EC_50_ values for NVR 3-778 against HBV DNA replication were within 3-fold of each other. While this is a limited panel of HBV isolates, the antiviral efficacy of NVR 3-778 has been demonstrated in humanized mice infected with GT C HBV (13) and in clinical studies in patients infected with GT A, B, or C HBV (unpublished data). The effect of the genotype-dependent susceptibility to NVR 3-778 or CAMs from other chemical classes would require further screening of additional HBV isolates that are replication competent and/or available from the serum of patients infected with different genotypes.

The preclinical antiviral activity and the absorption, distribution, metabolism, excretion, and safety profile of NVR 3-778 showed that antiviral efficacious concentrations of NVR 3-778 can be achieved in animals and support the further development of NVR 3-778 to identify safe and efficacious doses in HBV-infected patients. The target concentration of NVR 3-778 for efficacy was initially aimed at one that reached or exceeded plasma trough concentrations of 10-fold the antiviral EC_50_ value, or about 3 µM NVR 3-778. This target was confirmed in humanized mice, where all mice showed plasma trough concentrations of at least 3 µM and all mice responded to NVR 3-778 treatment with significant reductions in HBV DNA and secreted HBV RNA levels (13). Results from *in vitro* cell-based human protein binding studies showed that the EC_50_ of NVR 3-778 increased by about 16-fold in the presence of 40% human serum protein. The impact of serum protein binding on the antiviral activity of NVR 3-778 was further investigated using metabolically active primary human hepatocytes: a moderate increase in the EC_50_ (about 4-fold) for NVR 3-778 against HBV DNA replication was observed in the presence of serum proteins, which could be contributed by a reduction of the compound free fraction but also by an increase in compound stabilization against hepatic metabolism upon protein binding (25). In order to achieve at least such and higher plasma levels, the subsequent clinical program focused on twice-daily dosing to evaluate the optimal dosage for the efficacy of NVR 3-778 to maximize achievable trough levels of the drug (unpublished data), as guided by the results from preclinical study *in vitro* antiviral efficacies, human serum protein shift studies, and *in vivo* pharmacokinetic analysis.

## MATERIALS AND METHODS

### Compounds.

NVR 3-778 was synthesized by WuXi AppTec (Wuhan, China). Lamivudine, tenofovir, tenofovir disoproxil fumarate, and entecavir were purchased from Toronto Research Chemicals (Toronto, Canada).

### Electron microscopy.

Electron microscopy with HBV capsid formed from recombinant HBV core protein was performed as described previously (5). Samples were adsorbed on 200-mesh copper grids coated with Formvar carbon film and stained with fresh 2% uranyl acetate. Samples were visualized on an FEI Technai T12 transmission electron microscope equipped with a 2Kx2K AMT MegaPLUS ES 4.0 charge-coupled-device camera. Images were acquired at a magnification of ×105,000.

### Cell culture.

HepG2.2.15 cells (Fox Chase Cancer Center) containing GT D HBV were maintained in Dulbecco’s modified Eagle medium (DMEM) containing 10% fetal bovine serum (FBS), 380 μg/ml Geneticin, 2 mM l-glutamine, 100 units/ml penicillin, and 10 µg/ml streptomycin. HepG2 cells were obtained from the American Type Culture Collection and maintained in humidified incubators at 37°C with 5% CO_2_ in DMEM, 10% FBS, 100 units/ml penicillin, and 10 µg/ml streptomycin.

### HBV-containing plasmids.

Plasmid DNA containing 1.1 times the length of the genotype (GT) B HBV genome under the control of a CMV promoter (Fudan University, China) was previously cloned from the serum of an infected patient prior to LMV treatment (GenBank accession number AY220698) and after development of resistance to LMV (GenBank accession number AY220697) (26). Genotyping analysis confirmed that both isolates belonged to GT B HBV and that the isolate collected after the development of resistance to LMV contained both the L180M and M204V amino acid changes within the reverse transcriptase (rt) gene coding region (rtL180M/M204V). Plasmids containing HBV DNA with rtN236T, rtA181V, rtA181V/N236T, and rtL180M/M204V/N236T were generated using the GT B WT or rtL180M/M204V DNA-containing plasmids and site-directed mutagenesis (Agilent Technologies). A panel of nine different HBV core variants (cW102G, cW102R, cI105V, cI105L, cI105T, cT109S, cT109M, cT109I, and cY118F) was generated, also using the GT B WT DNA-containing plasmid and site-directed mutagenesis. The full-length genome from all plasmids was sequenced to confirm that only the intended nucleotide change(s) was present. Finally, HBV sequences from genotypes A (GenBank accession number AF305422), C (GenBank accession number AB033550), D (GenBank accession number V01460), E (GenBank accession number AB274971), F (GenBank accession number AB036912), G (GenBank accession number AF160501), and H (GenBank accession number AB375159) were gene synthesized as 1.1 times the genome length together with part of the CMV promoter at the 5′ end. The gene-synthesized piece was validated by sequencing and subcloned into the pCMV-HBV plasmid using the restriction sites SalI and NdeI (Genewiz, USA).

### Transient-transfection antiviral studies.

HepG2 cells were seeded in 96-well plates at a density of 20,000 cells/well and allowed to attach overnight at 37°C in 5% CO_2_. Cells were cotransfected with the HBV plasmids (100 ng/well) and a *Gaussia* expression plasmid (10 ng/well) (Thermo Scientific) using the Lipofectamine LTX Plus transfection reagent (Life Technologies). Transfection mixtures were removed after overnight incubation, and cells were treated with serially diluted NVR 3-778 at a final dimethyl sulfoxide (DMSO) concentration of 0.5%. After 3 days of incubation with the compound, the supernatants were collected and secreted *Gaussia* luciferase was measured using a *Gaussia* Flash luciferase assay kit (Thermo Scientific). Cells were lysed with 0.33% NP-40 (Thermo Scientific), and the cytoplasmic fractions were treated with 2 units of Turbo DNase (Invitrogen) and 10 units of S7 nuclease (Roche) in CutSmart buffer (New England Biolabs) containing 25 µM CaCl_2_ (G-Biosciences) at 37°C for 60 min, followed by inactivation of the enzymes at 75°C for 15 min. Samples were diluted into lysis buffer (Affymetrix) containing 2.5 µg proteinase K (Affymetrix) at 50°C for 40 min. Intracellular encapsidated HBV DNA was denatured for 30 min at 25°C in 0.2 M NaOH (Sigma) and incubated with HBV DNA probes designed to hybridize to the minus-strand HBV DNA (Affymetrix). The denatured DNA was subsequently detected using a QuantiGene assay kit, according to the branched DNA (bDNA) assay recommended by the manufacturer (Affymetrix).

### Antiviral studies using HepG2.2.15 cells.

HepG2.2.15 cells were seeded in 96-well plates at a density of 40,000 cells/well and incubated with serially diluted compounds at a final DMSO concentration of 0.5%. In combination studies, cells were treated with compounds either alone or in combination after combining the compounds in a checkerboard format. In studies examining the effect of human serum, serially diluted NVR 3-778 was added into cell culture medium containing 0% to 40% human serum (BioreclamationIVT). Cells were incubated with the compounds for 3 days, after which the medium was removed, fresh medium containing the compounds was added to the cells, and the cells were incubated for another 3 days prior to harvesting of the supernatants or cells for antiviral assays. To quantify intracellular encapsidated rcDNA or encapsidated pgRNA, cells were first solubilized using lysis buffer containing 1% NP-40 and 10 mM CaCl_2_. The cytoplasmic fraction was then treated with S7 nuclease (6 units) to remove nonencapsidated nucleic acids, after which the S7 nuclease was inactivated by the addition of EDTA (0.5 M). To quantify extracellular HBV DNA or HBV RNA, supernatants from HepG2.2.15 cells were treated with Turbo DNase at 37°C for 60 min, followed by inactivation of DNase at 75°C for 15 min. The levels of HBV DNA were determined by a bDNA assay as described above (Affymetrix). Supernatants or cell lysates containing HBV RNA were treated with proteinase K (2.5 µg) at 50°C for 40 min, transferred to a capture plate containing HBV RNA probe sets, and hybridized overnight at 55°C. Captured HBV RNA was measured by the addition of a chemiluminescent substrate using a QuantiGene assay (Affymetrix). To determine the cytotoxicity effect of the compounds on proliferating cells, HepG2.2.15 cells were seeded in 96-well plates at a density of 10,000 cells/well and incubated with serially diluted compounds at a final DMSO concentration of 0.5% for 3 days, after which the medium was removed, fresh medium containing the compounds was added to the cells, and the cells were incubated for another 3 days. At day 6, the CellTiter-Glo reagent was added to the cells and viability was determined according to the manufacturer’s protocol (Promega). Luminescence signals were measured at 0.2 s using a Victor X4 multimode plate reader (PerkinElmer).

### *De novo* infection study using PHH.

Cryopreserved PHH (Life Technologies) were seeded at 40,000 cells/well 1 day before infection. The HBV inoculum, concentrated from the supernatants of HepG2.2.15 cells, was prepared at 200 genome equivalents/cell in PHH medium (DMEM containing 10% FBS and a supplement cocktail containing HEPES, l-proline, insulin epidermal growth factor, dexamethasone, and ascorbic acid-2-phosphate, as previously described [10]) with 2% DMSO and 4% polyethylene glycol (PEG) 8000 (Sigma). Compounds were serially diluted in DMSO at 50-fold strength and added to PHH medium containing the HBV inoculum, prior to infecting the PHH. After overnight incubation, the infection mixtures were removed and the cells were replenished with PHH medium containing freshly diluted compounds. On day 5 and day 8 postinfection, the media containing the compounds were removed and the cells were replenished with PHH medium containing freshly diluted compounds. Supernatants and cells were harvested for antiviral and viability assays on day 11 postinfection. HBV DNA, HBsAg, and HBeAg were determined from the supernatants of infected PHH using bDNA assays (Affymetrix) and HBsAg or HBeAg chemiluminescence immunoassay kits (Autobio), as recommended by the manufacturers. Intracellular total HBV RNA mRNA and host mRNA levels were monitored by using the QuantiGene assays (Affymetrix) and probe sets that hybridized to the X region, which is common to all HBV transcripts, and the host β-actin mRNA, respectively.

### Data analysis.

To determine percent inhibition, the mean background signal from wells containing only culture medium was subtracted from the signal for all other samples, and percent inhibition at each compound concentration was calculated by normalization to the signals for HepG2.2.15 cells treated with 0.5% DMSO. EC_50_ values, effective concentrations that achieved a 50% inhibitory effect, were determined by nonlinear fitting using GraphPad Prism software (San Diego, CA). For the study of compound combinations, results from three independent plates were analyzed. Both CalcuSyn and MacSynergy II (Ann Arbor, MI) software were used to evaluate the effect of compound combinations (27). After data analysis at the 95% confidence level, values of synergy or antagonism of <25 µM^2^% were defined as insignificant, those between 25 and 50 µM^2^% were defined as minor, those between 50 and 100 µM^2^% were defined as moderate, and those of >100 µM^2^% were defined as strong.

### Pharmacokinetics.

*In vivo* pharmacokinetics studies were performed at Covance Laboratories (Madison, Wisconsin), accredited by the Association for Assessment and Accreditation of Laboratory Animal Care (AAALAC). All procedures in the protocols were in compliance with applicable animal welfare acts and were approved by the local Institutional Animal Care and Use Committee (IACUC). For the 28-day study in mice, male and female mice were 6 to 7 weeks old at the initiation of dosing. Environmental conditions, diet, and water were typical for pharmacokinetic and tolerability studies. NVR 3-778 was dosed in a vehicle of 0.5% (wt/vol) hydroxypropyl methylcellulose (Methocel E50 premium)--1% (wt/vol) polysorbate 80 (Tween 80) in reverse osmosis water. Blood samples were collected at 0.25, 0.5, 1, 3, 6, and 12 h after dosing on day 1 and on day 28 of the study into potassium (K_2_) EDTA. Plasma was harvested and stored at −80°C.

To determine oral bioavailability in dogs, two male beagle dogs were administered NVR 3-778, formulated in 1% DMSO--99% PEG 400 as a clear solution, by single intravenous bolus administration at 0.5 mg/kg. After a 7-day washout period, the same two dogs were administered NVR 3-778 by a single oral gavage of the same formulation at 1.5 mg/kg. Blood samples were collected at 0 (predose), 2 min (for the i.v. group only), 5 min, 15 min, and 0.5, 1, 3, 6, 9, and 24 h postdose. The concentration of NVR 3-778 in plasma samples was determined using LC-MS/MS methods.

## Supplementary Material

Supplemental file 1
