# Supplementary material for: Preclinical Characterization of NVR 3-778, a First-in-Class Capsid Assembly Modulator against Hepatitis B Virus
Source: Antimicrob Agents Chemother. 2018 Dec 21;63(1):e01734-18. doi: 10.1128/AAC.01734-18 (PMC6325219; doi:10.1128/AAC.01734-18)
Supplement: Supplemental file 1 [file d3642b80e5cf25af65e4d5e703598570_AAC.01734-18-s0001.pdf]

Preclinical Characterization of NVR 3-778, a First-in-Class Capsid Assembly Modulator Against the Hepatitis B Virus

Angela M Lam*, Christine Espiritu, Robert Vogel, Suping Ren, Vincent Lau, Mollie Kelly, Scott D Kuduk, George D Hartman, Osvaldo A Flores, Klaus Klumpp

Address: Novira Therapeutics Inc, part of the Janssen Pharmaceutical companies, 1400 McKean Road, Spring House, PA 19477, USA

SUPPLEMENTARY FIGURES AND TABLE

Figure S1. NVR 3-778 inhibits replication of HBV reverse transcriptase variants that are resistant to nucleos(t)ide analogs. (A) Relative replication capacities of HBV reverse transcriptase variants. Replication capacities of the resistant variants were normalized to wild-type (WT) HBV, which was set to 100% replication competent. HepG2 cells transfected with HBV plasmids containing either WT or reverse transcriptase variants were treated with increasing concentrations of NVR 3-778 or nucleos(t)ide analogs for 3 days. (B) Antiviral effect of NVR 3-778 against HBV reverse transcriptase variants L180M/M204V (▲), and L180M/M204V/N236T (LMMVNT) (■). (C) Antiviral effect of NVR 3-778 against A181V (▲), N236T (■) and A181V/N236T (AVNT) (♦). (D to F) Antiviral effect of LMV, ETV, or TDF against L180M/M204V (▲) and L180M/M204V/N236T (LMMVNT) (■) variants resistant to nucleos(t)ide analogs. (B to F) The dose response curves for NVR 3-778 and the nucleos(t)ide analogs against WT HBV (•) in each graph are shown as dashed lines. Data points represent mean values from at least three independent antiviral studies, and standard deviations are shown as error bars.

Figure S2. Antiviral activity of NVR 3-778 in PHH during *de novo* infection. (A) Cryopreserved PHH from three different donors were treated with media containing both HBV inoculum (200 GE/cell) and antiviral compounds (cpd). Increasing concentrations of (B, C) NVR 3-778 or (D, E) LMV were added into media containing HBV inoculum prior to the addition to PHH. Highest concentrations were set at 30 μM and compounds were 3-fold serially diluted for a total of 8 different concentrations. Compounds were replenished post infection three more times and cells were treated for a duration of 11 days prior to harvesting. (B, D) Levels of secreted HBV DNA (●), HBeAg (■), and HBsAg (▲) were monitored using supernatant from infected PHH. (C, E) Intracellular total HBV RNA (■) and beta-actin mRNA (●) were monitored from cell lysates using the Quantigene assay. Data points are mean values from experiments using three different PHH donors, with standard deviations shown as error bars.

Figure S3. Effect of human serum on NVR 3-778 antiviral activities. HepG2.2.15 cells were incubated with increasing concentrations of NVR 3-778 for 6 days in the presence of 0% (●), 10% (■), 20% (▲), or 40% (♦) human serum (HS). HBV DNA concentrations were determined from supernatant and compared to untreated cells containing the corresponding concentration of human serum. Data points represent mean values from at least three independent antiviral studies, and standard deviations are shown as error bars.

Table S1. HBV genotypes A to H used in transient transfection studies

GT	GenBank #	Genome length (bp)	Amino acid length				
			preC (N-ter)	C	Pol	preS/S	X
A	AF305422	3221	29	185	845	400	154
B	AY220698	3215	29	183	843	400	154
C	AB033550	3215	29	183	843	400	154
D	V01460	3182	29	183	832	389	154
E	AB274971	3212	29	183	842	399	154
F	AB036912	3215	29	183	843	400	154
G	AF160501	3248	2 stop codons	195	842	399	154
H	AB375159	3215	29	183	843	400	154

Figure S1

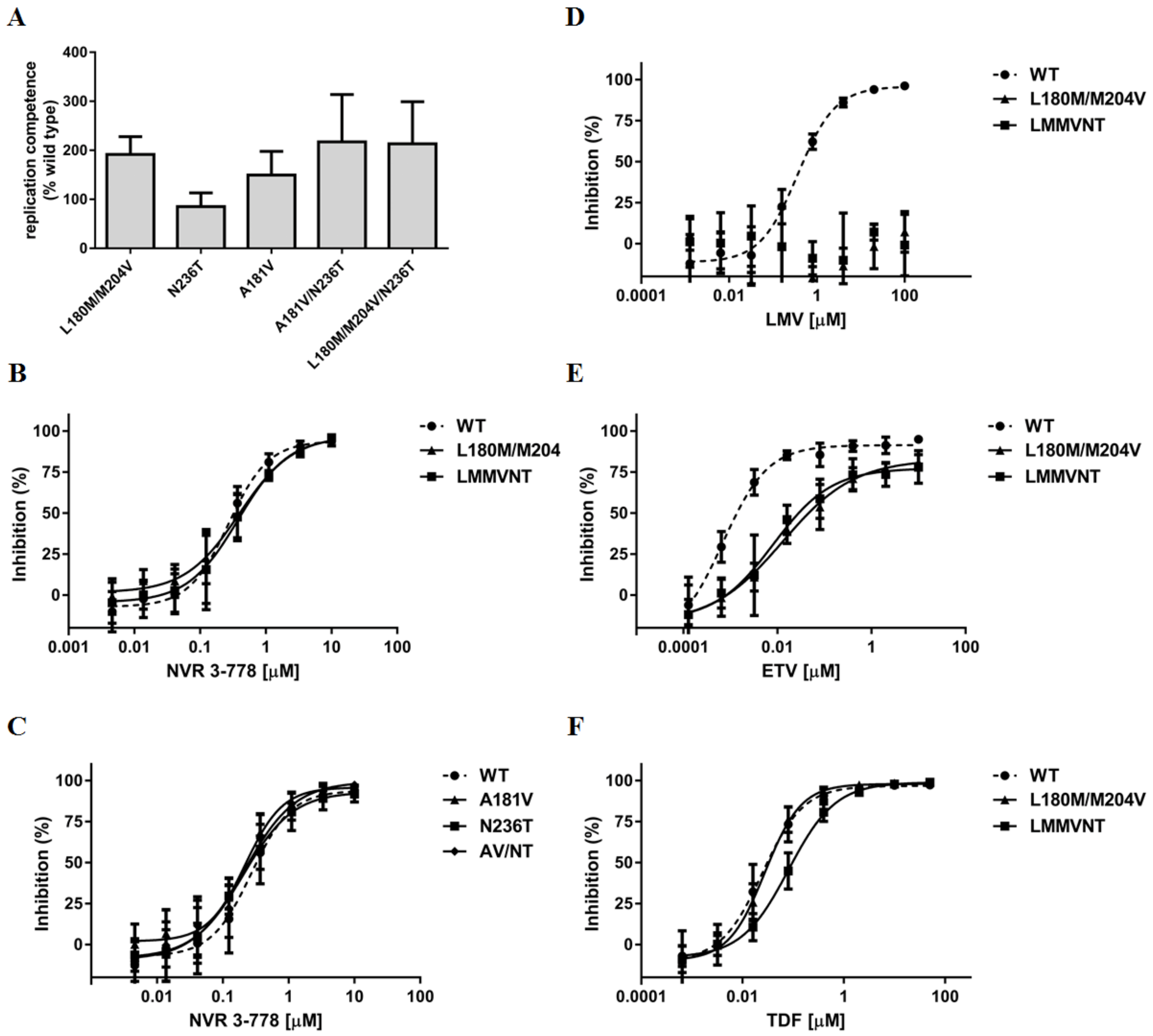
A
replication competence
(% wild type)
400
300
200
100
0
L180M/M204V
N236T
A181V
A181V/N236T
L180M/M204V/N236T
B
Inhibition (%)
100
75
50
25
0
0.001
0.01
0.1
1
10
100
NVR 3-778 [μM]
WT
L180M/M204
LMMVNT
C
Inhibition (%)
100
75
50
25
0
0.01
0.1
1
10
100
NVR 3-778 [μM]
WT
A181V
N236T
AV/NT
D
Inhibition (%)
100
75
50
25
0
0.0001
0.01
1
100
LMV [μM]
WT
L180M/M204V
LMMVNT
E
Inhibition (%)
100
75
50
25
0
0.0001
0.01
1
100
ETV [μM]
WT
L180M/M204V
LMMVNT
F
Inhibition (%)
100
75
50
25
0
0.0001
0.01
1
100
TDF [μM]
WT
L180M/M204V
LMMVNT

Figure S2

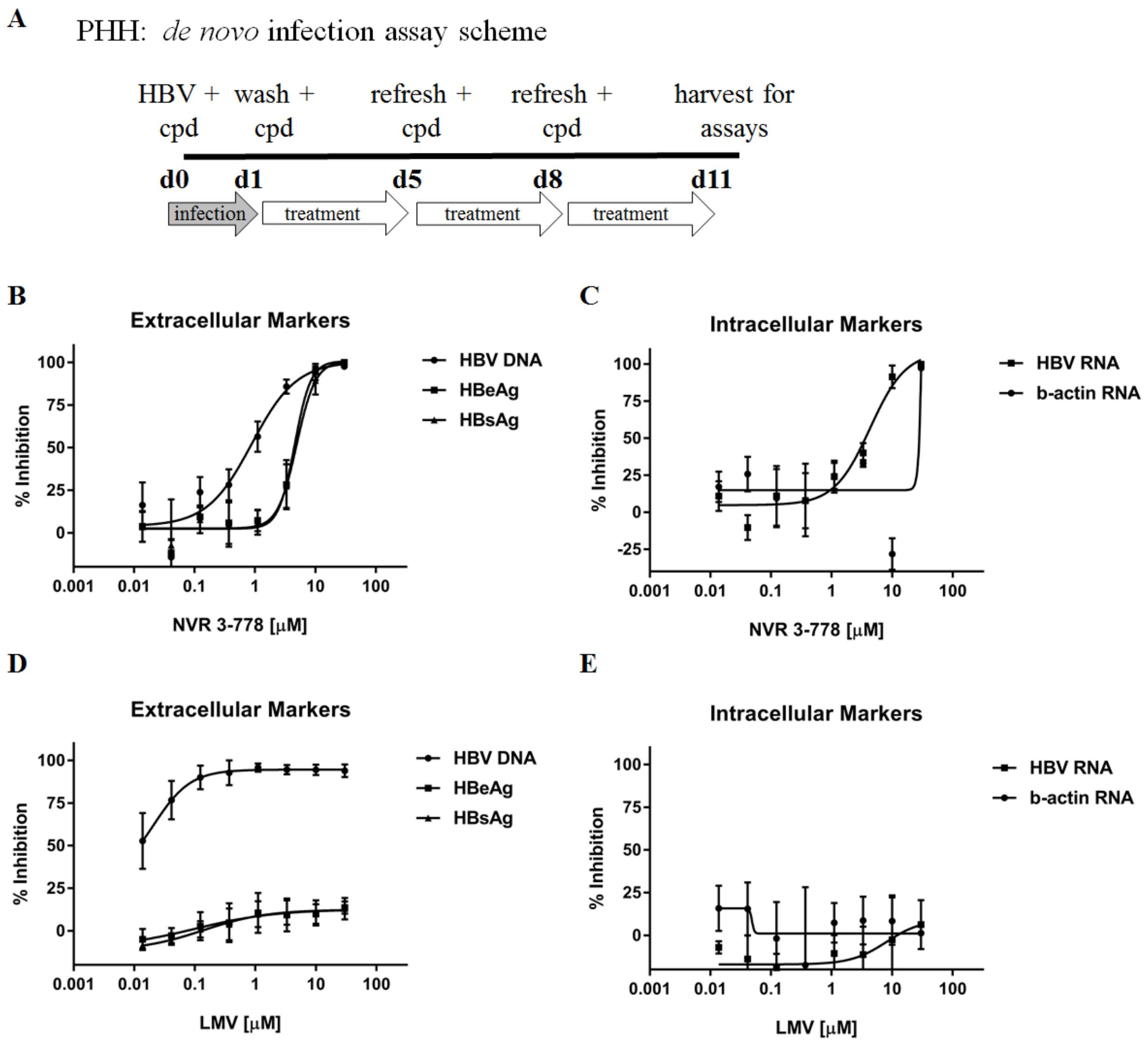
A
PHH: *de novo* infection assay scheme
HBV + cpd
wash + cpd
refresh + cpd
refresh + cpd
harvest for assays
d0
d1
d5
d8
d11
infection
treatment
treatment
treatment
B
Extracellular Markers
% Inhibition
100
75
50
25
0
0.001
0.01
0.1
1
10
100
NVR 3-778 [µM]
HBV DNA
HBeAg
HBsAg
C
Intracellular Markers
% Inhibition
100
75
50
25
0
-25
0.001
0.01
0.1
1
10
100
NVR 3-778 [µM]
HBV RNA
b-actin RNA
D
Extracellular Markers
% Inhibition
100
75
50
25
0
0.001
0.01
0.1
1
10
100
LMV [µM]
HBV DNA
HBeAg
HBsAg
E
Intracellular Markers
% Inhibition
100
75
50
25
0
0.001
0.01
0.1
1
10
100
LMV [µM]
HBV RNA
b-actin RNA

Figure S3

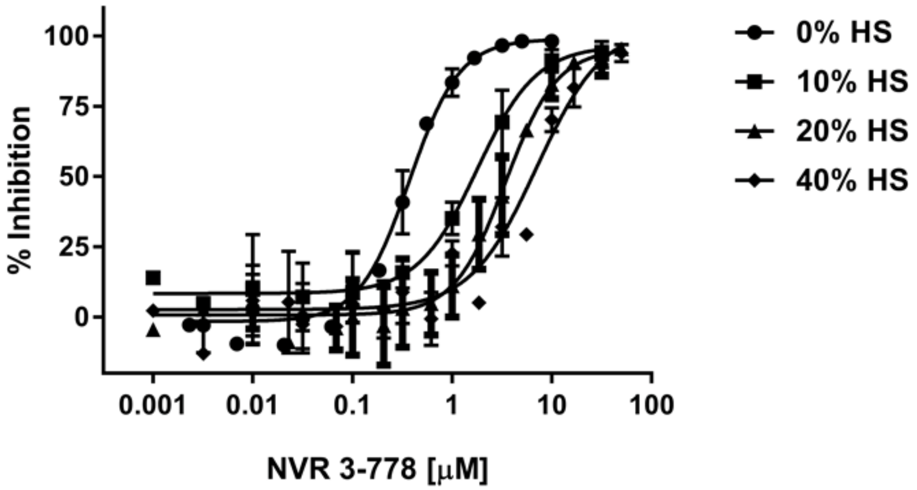
100
75
50
25
0
% Inhibition
0.001
0.01
0.1
1
10
100
NVR 3-778 [μM]
0% HS
10% HS
20% HS
40% HS
